# Supplementary material for: Candesartan Cilexetil In Vitro–In Vivo Correlation: Predictive Dissolution as a Development Tool
Source: Pharmaceutics. 2020 Jul 6;12(7):633. doi: 10.3390/pharmaceutics12070633 (PMC7408357; doi:10.3390/pharmaceutics12070633)
Supplement: Supplementary file 1 [file pharmaceutics-12-00633-s001.pdf]

pharmaceutics

MDPI

Supplementary Materials: Candesartan Cilexetil In Vitro–In Vivo Correlation: Predictive Dissolution as a Development Tool

Andrés Figueroa-Campos, Bárbara Sánchez-Dengra, Virginia Merino, Arik Dahan, Isabel González-Álvarez *, Alfredo García-Arieta, Marta González-Álvarez and Marival Bermejo

Berkeley Madonna code S1: IVIVC 1-step (A). Code for obtaining the predicted plasma profiles of candesartan cilexetil products from in vitro dissolution data, according to the model 1 described in the text. In this model, the link between *in vitro* dissolution and *in vivo* dissolution was hypothesized to be direct with a scaling function in time.

```
METHOD RK4

STARTTIME = 0 ; (h)
STOPTIME= 48 ; (h)
DT = 0.02 ; interval of times in which Berkeley Software iterates
rename TIME = t

Init(QdissR) = 0 ; initial amount of Reference dissolved in vitro (mg)
Init(QcR) = 0 ; initial amount of Reference in central compartment (mg)
Init(QpR) = 0 ; initial amount of Reference in peripheral compartment (mg)

Init(QdissA) = 0 ; initial amount of ProductA dissolved in vitro (mg)
Init(QcA) = 0 ; initial amount of ProductA in central compartment (mg)
Init(QpA) = 0 ; initial amount of ProductA in peripheral compartment (mg)

Init(QdissB) = 0 ; initial amount of ProductB dissolved in vitro (mg)
Init(QcB) = 0 ; initial amount of ProductB in central compartment (mg)
Init(QpB) = 0 ; initial amount of ProductB in peripheral compartment (mg)

aR = 1.9991 ;Reference a parameter (Weibull’s equation)
bR = 2.4987 ;Reference b parameter (Weibull’s equation)(h^a)
FmaxR = 96.9251 ;Reference Fmax parameter (Weibull’s equation) (%)

aA = 2.1649 ;ProductA a parameter (Weibull’s equation)
bA = 3.1969 ;ProductA b parameter (Weibull’s equation) (h^a)
FmaxA = 93.5042 ;ProductA Fmax parameter (Weibull’s equation) (%)

aB = 1.9748 ;ProductB a parameter (Weibull’s equation)
bB = 2.9052 ;ProductB b parameter (Weibull’s equation)(h^a)
FmaxB = 96.3768 ;ProductB Fmax parameter (Weibull’s equation) (%)

Dose = 32 ; (mg)
kel = 0.1143 ; elimination rate constant (h^-1)
k12 = 0.0668 ; central-to-peripheral rate constant (h^-1)
k21 = 0.1475 ; peripheral-to-central rate constant (h^-1)
Vc = 70 ; distribution volume (L)

m = 0.383
n = 0.161

tesc = m*t + n ; time scaling vitro-vivo equation (h)
```

```
QdissR' = (aR*FmaxR*(t^(aR-1))*exp((-(t^(aR)))/bR))/bR ; Reference in vitro
dissolution differential equation
QcR' = ((Dose*aR*(FmaxR/100)*(tesc^(aR-1))*exp((-(tesc^(aR)))/bR))/bR)-
(kel*QcR)-(k12*QcR)+(k21*QpR) ; Reference central compartment differential
equation
QpR' = (k12*QcR)-(k21*QpR) ; Reference peripheral compartment differential
equation

QdissA' = (aA*FmaxA*(t^(aA-1))*exp((-(t^(aA)))/bA))/bA; ProductA in vitro
dissolution differential equation
QcA' = ((Dose*aA*(FmaxA/100)*(tesc^(aA-1))*exp((-(tesc^(aA)))/bA))/bA)-
(kel*QcA)-(k12*QcA)+(k21*QpA) ; ProductA central compartment differential
equation
QpA' = (k12*QcA)-(k21*QpA) ; ProductAperipheral compartment differential
equation

QdissB' = (aB*FmaxB*(t^(aB-1))*exp((-(t^(aB)))/bB))/bB; ProductB in vitro
dissolution differential equation
QcB' = ((Dose*aB*(FmaxB/100)*(tesc^(aB-1))*exp((-(tesc^(aB)))/bB))/bB)-
(kel*QcB)-(k12*QcB)+(k21*QpB) ; ProductB central compartment differential
equation
QpB' = (k12*QcB)-(k21*QpB) ; ProductBperipheral compartment differential
equation

CpR = QcR/Vc ; (mg/L)
CpA = QcA/Vc ; (mg/L)
CpB = QcB/Vc ; (mg/L)
```

Berkeley Madonna code S2: IVIVC 1-step (B). Code for obtaining the predicted plasma profiles of candesartan cilexetil products from in vitro dissolution data, according to the model 2 described in the text. In this model, the *in vitro* parameter b from Weibull equation was scaled for the *in vivo* dissolution equation (b_{esc}) and an extra scaling factor (ESC) was introduced to capture the differences between the *in vitro* dissolution and the *in vivo* absorption.

```
METHOD RK4

STARTTIME = 0.001 ; (h)
STOPTIME= 48 ; (h)
DT = 0.02 ; interval of times in which Berkeley Software iterates
rename TIME = t

Init(QdissR) = 0 ; initial amount of Reference dissolved in vitro (mg)
Init(QdissescR) = 0 ; initial amount of Reference dissolved in vivo (mg)
Init(QcR) = 0 ; initial amount of Reference in central compartment (mg)
Init(QpR) = 0 ; initial amount of Reference in peripheral compartment (mg)

Init(QdissA) = 0 ; initial amount of ProductA dissolved in vitro (mg)
Init(QdissescA) = 0 ; initial amount of ProductA dissolved in vivo (mg)
Init(QcA) = 0 ;initial amount of ProductA in central compartment (mg)
Init(QpA) = 0 ; initial amount of ProductA in peripheral compartment (mg)

Init(QdissB) = 0 ; initial amount of ProductB dissolved in vitro (mg)
Init(QdissescB) = 0 ; initial amount of ProductB dissolved in vivo (mg)
Init(QcB) = 0 ; initial amount of ProductB in central compartment (mg)
Init(QpB) = 0 ; initial amount of ProductB in peripheral compartment (mg)

aR = 1.9991 ;Reference a parameter (Weibull’s equation)
bR = 2.4987 ;Reference b parameter (Weibull’s equation)(h^a)
FmaxR = 96.9251 ;Reference Fmax parameter (Weibull’s equation) (%)

aA = 2.1649 ;ProductA a parameter (Weibull’s equation)
bA = 3.1969 ;ProductA b parameter (Weibull’s equation)(h^a)
FmaxA = 93.5042 ;ProductA Fmax parameter (Weibull’s equation) (%)

aB = 1.9748 ;ProductB a parameter (Weibull’s equation)
bB = 2.9052 ;ProductB b parameter (Weibull’s equation)(h^a)
FmaxB = 96.3768 ;ProductB Fmax parameter (Weibull’s equation) (%)

Dose = 32 ; (mg)
kel = 0.1143 ; elimination rate constant (h^-1)
k12 = 0.0668 ; central-to-peripheral rate constant (h^-1)
k21 = 0.1475 ; peripheral-to-central rate constant (h^-1)
Vc = 70 ; distribution volume (L)

m = 2.611
n = -0.420

bResc = (n + m*(bR^(1/aR)))^(aR) ; Reference b parameter scaling vitro-vivo
equation (h^a)
bAesc = (n + m*(bA^(1/aA)))^(aA) ; ProductA b parameter scaling vitro-vivo
equation (h^a)
bBesc = (n + m*(bB^(1/aB)))^(aB) ; ProductB b parameter scaling vitro-vivo
equation (h^a)

ESC = IF t<=0.5 THEN (u1*t + v1) ELSE IF (t>0.5 AND t<=2) THEN (u2*t + v2) ELSE
IF (t>2 AND t<=4.5) THEN (u3*t + v3) ELSE IF (t>4.5 AND t<=10) THEN (u4*t + v4)
ELSE (u5*t + v5) ; in vitro dissolution-absorption scaling factor
```

```
u1 = -3.4102
v1 = 0
u2 = 6.6897
v2 = -5.6314
u3 = -5.2760
v3 = 20.6963
u4 = -1.4830
v4 = 0.1902
u5 = 0.3141
v5 = -17.4432

QdissR' = (aR*FmaxR*(t^(aR-1))*exp((-(t^(aR)))/bR))/bR; Reference in vitro dissolution differential equation
QdissescR' = (Dose*aR*(FmaxR/100)*(t^(aR-1))*exp((-(t^(aR)))/bResc))/bResc; Reference in vivo dissolution differential equation
QcR' = (ESC+QdissescR')-(kel*QcR)-(k12*QcR)+(k21*QpR) ; Reference central compartment differential equation
QpR' = (k12*QcR)-(k21*QpR) ; Reference peripheral compartment differential equation

QdissA' = (aA*FmaxA*(t^(aA-1))*exp((-(t^(aA)))/bA))/bA ; ProductA in vitro dissolution differential equation
QdissescA' = (Dose*aA*(FmaxA/100)*(t^(aA-1))*exp((-(t^(aA)))/bAesc))/bAesc ; ProductA in vivo dissolution differential equation
QcA' = (ESC+QdissescA')-(kel*QcA)-(k12*QcA)+(k21*QpA) ; ProductA central compartment differential equation
QpA' = (k12*QcA)-(k21*QpA) ; ProductAperipheral compartment differential equation

QdissB' = (aB*FmaxB*(t^(aB-1))*exp((-(t^(aB)))/bB))/bB; ProductB in vitro dissolution differential equation
QdissescB' = (Dose*aB*(FmaxB/100)*(t^(aB-1))*exp((-(t^(aB)))/bBesc))/bBesc; ProductB in vivo dissolution differential equation
QcB' = (ESC+QdissescB')-(kel*QcB)-(k12*QcB)+(k21*QpB) ; ProductB central compartment differential equation
QpB' = (k12*QcB)-(k21*QpB) ; ProductBperipheral compartment differential equation

CpR = QcR/Vc ; (mg/L)
CpA = QcA/Vc ; (mg/L)
CpB = QcB/Vc ; (mg/L)
```

Table S1. Initial and final values for the parameters of the two models used for obtaining one-step IVIVCs. R, A and B referes to Reference, product A and product B respectively.

**MODEL 1 - t_{esc} IVIVC**				**MODEL 2 - b_{esc} and ESC IVIVC**			
**Parameter (Units)**	**Initial**	**Final**		**Parameter (Units)**	**Initial**	**Final**	
a_R	1.9991	2.0003	*	a_R	1.9991	1.9991	
b_R (h^a)	2.4987	2.4998	*	b_R (h^a)	2.4987	2.4987	
$Fmax_R$ (%)	96.9251	96.9234	*	$Fmax_R$ (%)	96.9251	96.9251	
a_A	2.1649	2.1655	*	a_A	2.1649	2.1649	
b_A (h^a)	3.1969	3.1959	*	b_A (h^a)	3.1969	3.1969	
$Fmax_A$ (%)	93.5042	93.4806	*	$Fmax_A$ (%)	93.5042	93.5042	
a_B	1.9748	1.9751	*	a_B	1.9748	1.9748	
b_B (h^a)	2.9052	2.9050	*	b_B (h^a)	2.9052	2.9052	
$Fmax_B$ (%)	96.3768	96.3777	*	$Fmax_B$ (%)	96.3768	96.3768	
Dose (mg)	32	32		Dose (mg)	32	32	
k_{el} (h^{-1})	0.1143	0.1143		k_{el} (h^{-1})	0.1143	0.1143	
k_{12} (h^{-1})	0.0668	0.0668		k_{12} (h^{-1})	0.0668	0.0668	
k_{21} (h^{-1})	0.1475	0.1475		k_{21} (h^{-1})	0.1475	0.1475	
V_c (L)	70	220.704	*	V_c (L)	70	70	
m	0.383	0.383		m	2.611	2.611	
n	0.161	0.161		n	-0.420	-0.420	
				u_1	-3.4102	-2.6258	*
				v_1	0.0000	-0.2634	*
				u_2	6.6897	5.9416	*
				v_2	-5.6314	-4.2030	*
				u_3	-5.2760	-2.6593	*
				v_3	20.6963	6.0413	*
				u_4	-1.4830	0.0101	*
				v_4	0.1902	0.0000	*
				u_5	0.3141	0.0055	*
				v_5	-17.4432	-0.1333	*

* indicates that the parameter was adjusted

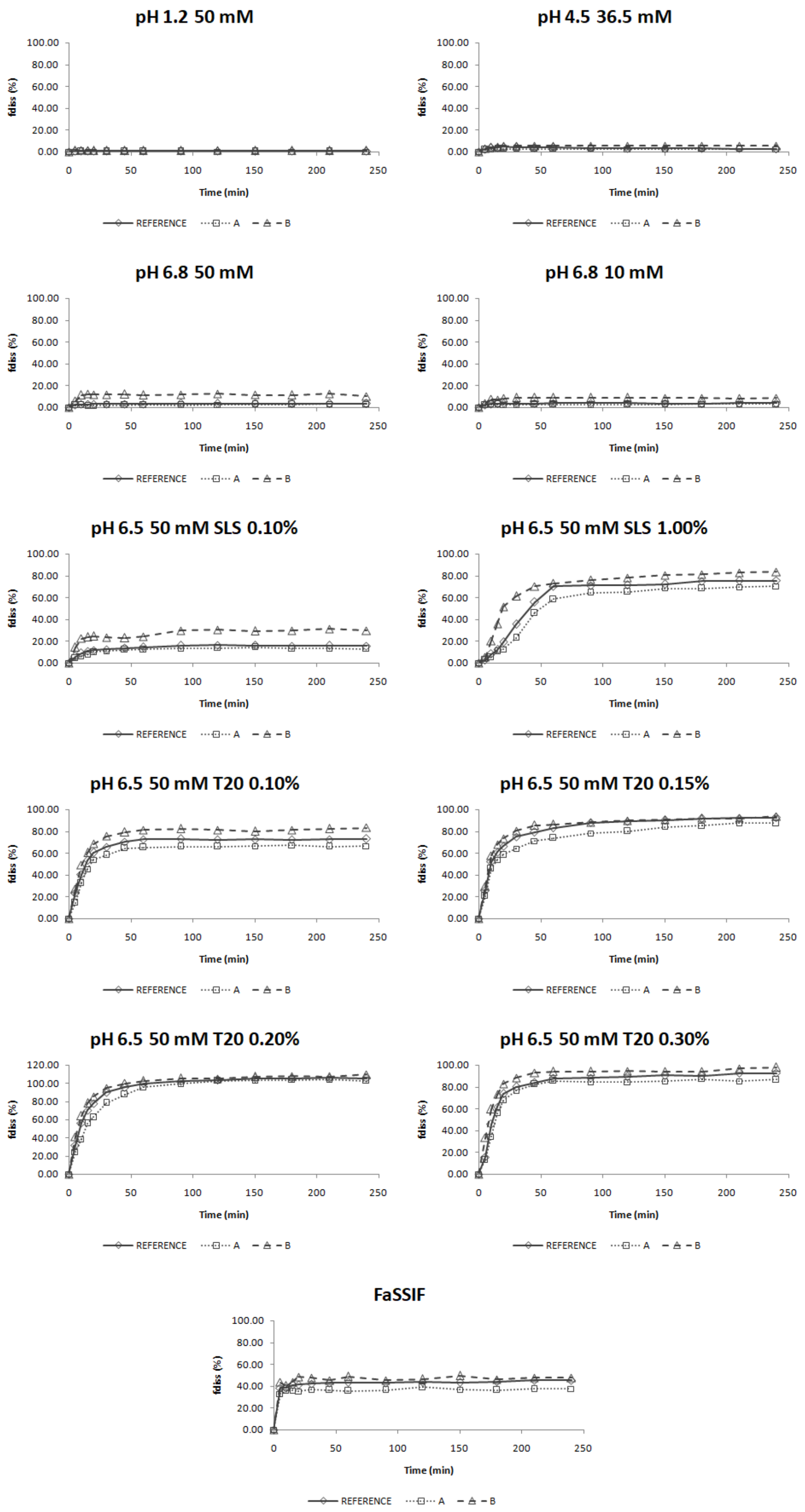

Figure S1. Dissolution profiles of the three products of Candesartan cilexetil (Reference, Product A and Product B) obtained in different conditions in USP II apparatus. SLS = Sodium Lauryl Sulfate, T20 = Tween 20, fdiss = fraction dissolved
